# Management of Type 2 Diabetes Mellitus through Telemedicine

**DOI:** 10.1371/journal.pone.0126858

**Published:** 2015-05-14

**Authors:** Claudio Carallo, Faustina Barbara Scavelli, Maurizio Cipolla, Valentina Merante, Valeria Medaglia, Concetta Irace, Agostino Gnasso, Progetto Diabete Calabria

**Affiliations:** 1 Metabolic Diseases Unit, Department of Clinical and Experimental Medicine, “Mater Domini” Hospital, “Magna Græcia” University, Catanzaro, Italy; 2 General Practitioners, National Health Service, Catanzaro, Italy; National University of Singapore, SINGAPORE

## Abstract

**Background:**

Type 2 diabetes mellitus T2DM has a huge and growing burden on public health, whereas new care models are not implemented into clinical practice; in fact the purpose of this study was to test the effectiveness of a program of integrated care for T2DM, compared with ordinary diligence.

**Methods:**

"Progetto Diabete Calabria" is a new organizational model for the management of patients with diabetes mellitus, based on General Practitioners (GPs) empowerment and the use of a web-based electronic health record, shared in remote consultations among GPs and Hospital Consultants. One-year change in glucose and main cardiovascular risk factors control in 104 patients (Cases) following this integrated care program has been evaluated and compared with that of 208 control patients (Controls) matched for age, gender, and cardiometabolic profile, and followed in an ordinary outpatient medical management by the Consultants only. Both patient groups had Day Hospitals before and after the study period.

**Results:**

The mean number of accesses to the Consultants during the study was 0.6±0.9 for Cases, and 1.3±1.5 for Controls (p<0.0001). At follow-up, glycated hemoglobin (HbA1c) significantly decreased from 58±6 to 54±8 mmol/mol in Cases only (p=0.01); LDL cholesterol decreased in both groups; body mass index decreased in Cases only, from 31.0±4.8 to 30.5±4.6 kg/m^2^ (p=0.03).

**Conclusions:**

The present study demonstrates that a health care program based on GPs empowerment and taking care plus remote consultation with Consultants is at least as effective as standard outpatient management, in order to improve the control of T2DM.

## Introduction

Type 2 diabetes mellitus (T2DM) is one of the most frequent metabolic disorders. In Italy, estimated prevalence is 4.9% on a national basis [1], with peaks of 10% in Southern regions [2]. Diabetes incidence increases with age, and among subjects ≥75 years one out of five has this alteration. Its burden on healthcare costs, both direct (diagnosis and therapy of the disease and its complications) and indirect (reduction of work capacity, need for assistance with situations such as blindness, amputations and hemodialysis) is huge and growing [3].

The prevention or delay of the onset of the disease and its complications is expensive and requires the rationalization of the diagnostic and therapeutic tools available. In chronic diseases such as diabetes mellitus, the repetition of blood and instrumental examinations, overlapping of diagnostic and therapeutic decisions among different caregivers, and finally inappropriate hospitalization are frequent. To this regard, the World Health Organization recommends the implementation of integrated care models [4]. In Italy, some programs for the integrated management of T2DM have been tested, after adequate formation and training of the General Practitioners (GP) [5,6]. Unfortunately, this model has not been widely implemented into clinical practice.

Starting from these evidences, "Project for Diabetes in Calabria" (PDC), a program of integrated care to patients with T2DM in the South of Italy, was held from 2010 to 2014, including GPs empowerment and the use of a web-based electronic health record, shared in teleconsultation among GPs and Hospital Consultants.

Aim of the study was to verify the efficacy of this model with respect to the ordinary care in a clinical setting. To this end, all patients followed jointly by GPs and Consultants (Cases) were compared to twice as many patients (Controls) visited exclusively by Consultants.

## Patients and Methods

### Project outline

The research conforms to the ethical guidelines of the Declaration of Helsinki as reflected in a priori approval by the Ethical Committee of "Azienda Ospedaliera Mater Domini" (Catanzaro, Italy). All recruited subjects gave written informed consent.

PDC was a project developed from December 2010 to April 2014, and it was held in two steps:

- specific training on type 2 diabetes mellitus given by Consultants to the GPs, organized in Continuing Medical Education (CME) courses, producing written and agreed diagnostic and therapeutic pathways integrating National and International specific Guidelines;
- shared clinical management of the patients via medical data exchange among GPs and Consultants, by the connection of the respective electronic health record systems. Participants were screened for complications and given therapeutic advices by Hospital Consultants during a Day Hospital, and then controlled every year. GPs visited quarterly the participants, eventually in remote video- or voice-consultation with Hospital Consultants.

The participation in this project of patients, Consultants and GPs was on a voluntary basis.

### Set-up period

PDC was organized by the two Consultants working in the Metabolic Unit of the University Hospital, and by a GP coordinating a local GPs clinical association. All other GPs clinical association of the Province of Catanzaro were informed; five of them, including 32 GPs, agreed to participate to the first step of the project. Shortly after, another neighbouring GPs association and a local GP working not in a medical team, asked to participate. Therefore, the whole GPs number who participated into the first step was 43.

### Step one

#### Training section

Four one-day CME meetings were held concerning: prediabetes and diabetes onset; T2DM management by GPs; role for GPs in insulin therapy; telemedicine approach in T2DM. The topics within the training sections were chosen also upon needs expressed by the GPs via a questionnaire. At the end of the training, 33 GPs agreed to continue the project; a round table with the Consultants served to point out diagnostic and therapeutic pathways integrating National [5–7] and International [8–10] specific Guidelines. Those pathways took into account specificities of the geographical area in which the project fell (e.g. availability to perform oral glucose load; reliability of the glycosylated hemoglobin assay; choice of procedures for the determination of microalbuminuria).

### Step two

#### Preliminary phase

In order to start patients recruitment, supporting material explaining the project was prepared: flyers, a poster exposed in each GPs waiting room, a dedicated telephone number for the participants. After a speech with their GP, participants signed a written informed consent, receiving a unique code number identifying both patient and GP; by this code only, participants placed a phone reservation to hold their first Day Hospital. Booking system, used also for the other visits of the participant during the project, was based on a dedicated computer program capable to extract various statistics, as the timeline of reservations for each participant or for each GP. Consultants had a web based self-developed electronic health record, and half of the GPs had a different self-developed electronic system sharing, in real time, participant-specific clinical data with the Hospital system.

#### Clinical phase

Per protocol, participants had an annual Day Hospital at the Metabolic Unit of University Hospital, addressed to the evaluation of glycemic control and complications, and received therapeutic advices for major risk factors for atherosclerosis such as hypertension, obesity, dyslipidemia, smoking, alcohol abuse, unsafe lifestyle. Between these assessments, participants had at least quarterly visits by GPs, eventually in remote consultation with Hospital Consultants. GPs could request for any lab assessment and prescribe any therapy they needed, on an outpatient basis, also beyond those diagnostic and therapeutic pathways agreed with the Consultants at the study start.

GPs could address the participant to the Consultant by a telephone contact outside agreed accesses, in case of severe and / or repeated hypoglycaemia, rapidly deteriorating complications (cerebral, coronary, or peripheral vasculopathy), diabetic foot, appearance of leg ulcers, pregnancy, severe metabolic decompensation (blood glucose> 350 mg/dL, ketonuria, dehydration from osmotic polyuria). Each participant received during the Day Hospital a leaflet containing personalized desirable values of glycosylated hemoglobin, blood pressure, body weight, LDL cholesterol and triglycerides. This information was reinforced at the following visits.

#### Control arm

As a normal clinical organization for chronic outpatients at the University Hospital, patients with diabetes receive one annual Day Hospital and, if needed, quarterly ambulatory visits, all by the Consultants of the University Hospital. GPs are informed by Consultants through a letter directly delivered to the patient, but GPs usually do not participate in any management decision. Patients following this clinical care path, and who matched cases for age, gender, diabetes status and therapy, and common cardiometabolic risk factors, were enrolled as controls, after signing the informed consent. They were recruited into the same time period of the participants enrolled for the telemedicine project. Anthropometric measurements and laboratory analyses derived from the same equipments used for the participants in the project.

### Clinical and biochemical parameters

Examinations were performed as previously indicated [11]. Briefly, all participants in the study underwent complete clinical examination and blood tests at baseline and follow-up. Body mass index (BMI) was computed as weight (in Kg) divided by height (in m) squared. Systolic (SBP) and diastolic (DBP) blood pressures were measured, on the right arm, after the participant had been resting for at least 5 min, with a standardized sphygmomanometer.

Venous blood for biochemical analyses was collected after overnight fasting. Blood glucose, glycated hemoglobin (HbA1c), microalbuminuria and lipids were measured by routine methods. Diabetes was defined as fasting blood glucose ≥ 126 mg/dl and/or use of antidiabetic agents. Hyperlipidemia was defined as total cholesterol and/or triglycerides exceeding 200 mg/dl and/or use of lipid lowering drugs. Hypertension was defined as SBP/DBP ≥ 140/90 mmHg and/or use of antihypertensive agents.

### Statistics

Triglycerides, not normally distributed, were log transformed. All other continuous variables had normal distribution. In order to compare baseline features among cases and controls, Student’s t test for unpaired data was used for continuous variables, and chi-square for categorical values. The parameters changes after one year of follow-up within each group were evaluated by Student’s t test for paired data. Data was analysed using SPSS v. 17.0 software.

## Results

Patients recruitment started in late April 2011; after 3 years, 566 participants were addressed to the Consultants by the 25 participating GPs, but 71 (13%) missed their appointment. During the first visit by the Consultants, 85 (17%) were not included because not affected by diabetes or refusing signing the consent. Among the 410 remaining, 380 only got their appointment for the first Day Hospital. Among them, 114 participants had their second Day Hospital also, within the time chosen for the database lock; after the exclusion of 10 participants suffering from major cardiovascular disease, 104 patients with diabetes were recruited for the present case control study. Mean time between the two Day Hospitals was 15.1±0.9 months.

In the same period, 208 patients with diabetes without major cardiovascular diseases, followed by the same Operative Unit in the ordinary medical management, and matching Cases for age, gender, diabetes status and therapy, and common cardiometabolic risk factors were enrolled. These patients received two Day Hospitals, and in between quarterly visits by the Consultants. Mean time between the two Day Hospitals in this group was 15.3±1.0 months.

All baseline characteristics of Cases and Controls were similar (Table 1). The number of accesses to the Consultants between Day Hospitals was 0.6±0.9 in cases in remote consultations with the GPs, and 1.3±1.5 in controls in the usual outpatients Consultant facilities (p<0.0001). Mean duration of remote consultation was 7±3 minutes, whereas outpatient visit lasted 24±11 minutes.

**Table 1:** Baseline features of the two study groups.

	*CASES*	*CONTROLS*	* *p =*
*Male gender (%)*	63	62	ns
*Age (years)*	63.9 ± 9.3	61.4 ± 11.2	ns
*Body mass index (Kg/m2)*	31.0 ± 4.8	30.6 ± 5.8	ns
*Waist (cm)*	102.2 ±13.2	103.1 ± 13.2	ns
*Hypertension (%)*	77	74	ns
*Systolic blood pressure (mmHg)*	144.4 ±17.6	140.8 ± 18.1	ns
*Diastolic blood pressure (mmHg)*	84.5 ± 8.6	83.7 ± 11.3	ns
*Heart rate (bpm)*	74.4 ± 12.4	75.4 ± 12.1	ns
*Treatment of hypertension (%)*	70	66	ns
*Dyslipidemia (%)*	63	64	ns
*Total cholesterol (mg/dL)*	179.2 ±44.3	184.9 ± 47.5	ns
*LDL cholesterol (mg/dL)*	101.7 ±36.9	107.5 ± 40.6	ns
*HDL cholesterol (mg/dL)*	48.2 ± 11.9	47.4 ± 12.1	ns
*Triglycerides (mg/dL)*	140.5 ±74.5	147.7±106.2	ns
*Therapy for dyslipidemia (%)*	57	46	ns
*Onset of diabetes (years)*	8.3 ± 8.1	9.7 ± 8.6	ns
*Antidiabetic drugs (number)*	1.2 ± 0.8	1.3 ± 0.9	ns
*Insulin regimen (%)*	24	29	ns
*Microalbuminuria (mg/L)*	43 ± 94.8	62.2 ± 23.7	ns
*Glycemia (mg/dL)*	160.4 ±56.9	156.1 ± 54.1	ns
*Capillary blood glucose before breakfast (mg/dL)*	140.4 ±39.6	143.9 ± 39.5	ns
*Capillary blood glucose before lunch (mg/dL)*	131.9 ±39.6	132.4 ± 44.3	ns
*Capillary blood glucose after lunch (mg/dL)*	158.7 ±49.6	158 ± 47.9	ns
*Capillary blood glucose before dinner (mg/dL)*	139.4 ±44.3	138.5 ± 45	ns
*Capillary blood glucose after dinner (mg/dL)*	168.5 ±47.5	165.3 ± 47.9	ns
*HbA1c (mmol/mol)*	58 ± 6	61 ± 7	ns

Values are mean ± standard deviation unless otherwise indicated;

*statistical significance on t-test or chi-square;

ns = not significant.

**Fig 1:**
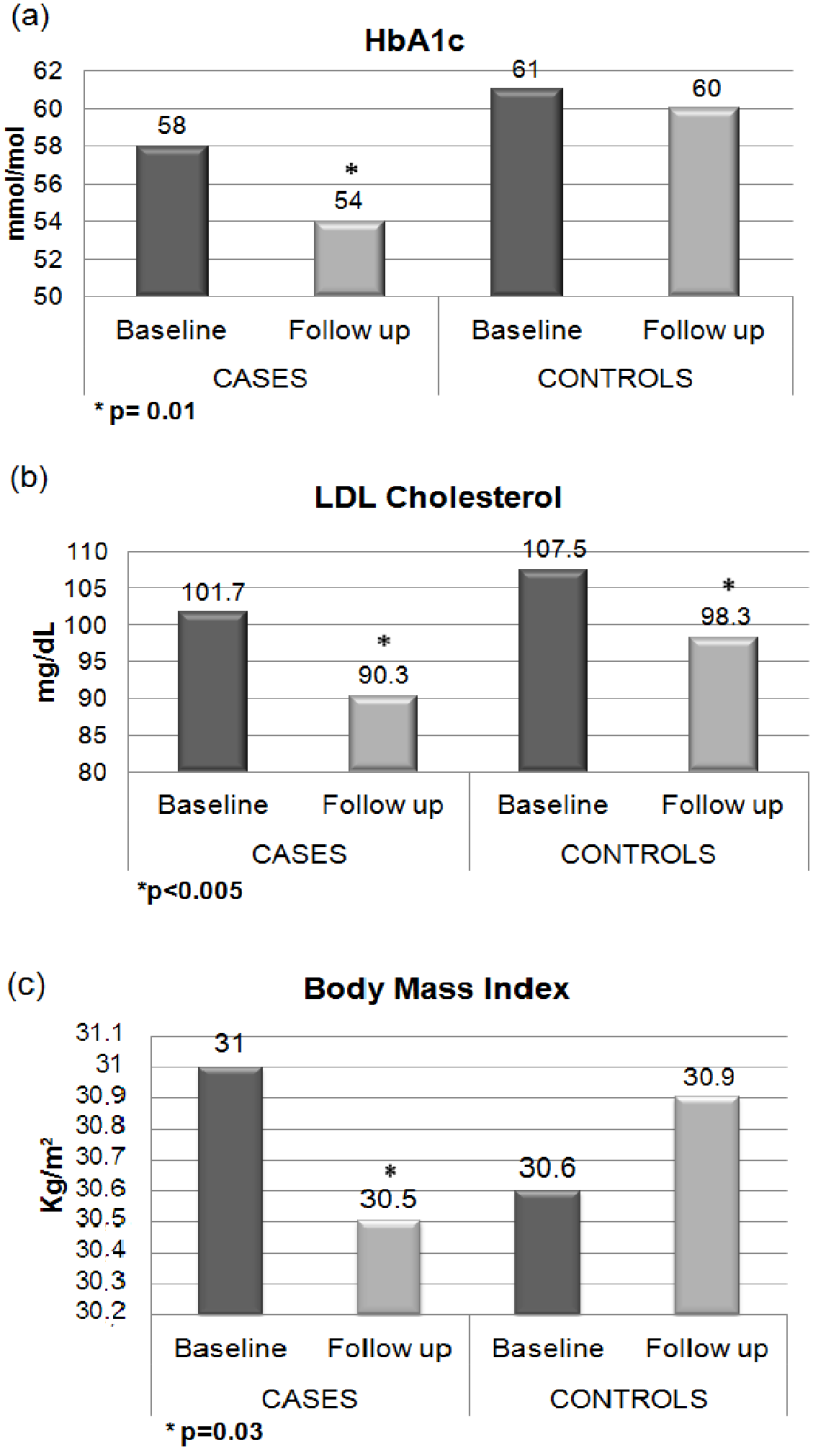
Variations in glycemic, lipidic and body fat indexes within cases and controls during the study project.

At follow up, Cases consistently improved diabetic profile and some other cardiometabolic risk factors; these results were similar or better than the achievements of the control group (Fig 1). In fact, at follow-up, HbA1c significantly decreased from 58±6 to 54±8 mmol/mol in Cases only (p = 0.01). LDL cholesterol decreased from 101.7±36.9 to 90.3±34.4 mg/dL in Cases (p = 0.003), and from 107.5±40.6 to 98.3±37.7 mg/dL in Controls (p = 0.001). BMI decreased in Cases only, from 31.0±4.8 to 30.5±4.6 kg/m^2^ (p = 0.03). Blood pressures, triglycerides, and waist were not significantly changed during the study both in Cases and in Controls (data not shown).

## Discussion

The present project has tested a remote cooperation among trained GPs and Hospital Consultants, by information technology and web communications. The results clearly demonstrate that GPs empowerment plus remote consultations are at least as effective as standard outpatient facilities, in order to improve cardiometabolic profile in T2DM.

Furthermore, the project had some positive effects beyond measured data, linked to the empowerment and to the update in clinical practice of the GPs. For example, as previously reported, a subgroup of 17 GPs participating into step one, who had in charge over 23000 subjects, after 18 months reported a prevalence of diabetes, hypertension, and dyslipidemia in that population, increased respectively by 13%, 12%, and 42% (2).

In times of budget restrictions, healthcare decision makers have high expectations on telemedicine, in terms of improving access to health care, overcoming Consultants shortage, and reducing health care costs while improving quality. Despite of this, organizational models are still matter of several investigations.

Previous studies have also demonstrated the importance of telemedicine in the integrated management of patients with diabetes.

A review summarized the potential benefits provided to the Physicians and patients by electronic instruments in the diabetes treatment [12]. This study found that telemedicine could: reduce the time spent by Doctors for therapeutic adjustments; improve the quality and quantity of data used to monitor the patient; increase the number of patients undergoing follow-up, without reducing the quality of care; optimize the number of visits of the Patient in the Hospital, providing them at the same time the best advice by Doctors.

A paper has described a protocol similar to the present study [13], but in a limited number of Medical Doctors and patients. There, it is proposed an integrated management of patients with T2DM through videoconferencing, between one Consultant and 4 GPs. They recruited 154 patients with diabetes followed for 12 months; Consultant was contacted 94 times. The study demonstrated the feasibility of therapeutic adjustments in video conferencing and reducing the number of Hospital admissions, improving the quality of care. Metabolic and hemodynamic parameters were significantly improved in the course of the study: HbA1c decreased from 8.1% to 7.8%, systolic blood pressure from 156 to 148 mm Hg and diastolic blood pressure from 88 to 83 mmHg.

The results of the present study expand these earlier observations in terms of study design, size of personnel, patients, and follow up length, confirming also that telemedicine might be a feasible and useful way to involve GPs into chronic disease management.

Indeed, an optimization of roles and commitments seems to positively derive from this care model. The significant increase in knowledge in the medical field, grown logarithmically in the last four decades [14], makes it difficult to upgrade GPs already heavily engaged in the care of patients [15]. As a consequence, GPs adherence to Evidence Based Medicine for the management of chronic disease is poor, and the patients receive only partially the recommended process of care [16]. In the proposed care model, hospital Consultants have the engagement to select GPs, update disease management according to the most recent guidelines, and control its application patient by patient together with the GPs. Aside from non-inferiority of the model, consultants save probably time to devote to complicated patients, and patients save certainly time and money by consulting their local GP instead of moving to the hospital outpatients facilities, often far away.

Further developments in the field of diabetic control might be added by the recent development of mobile health technologies, as for example by wearable glucose sensors [17]. This level or care might add a home care level to the present care model.

### Limitations

The present study design has some limitations.

Participation into the study was on a voluntary basis both for physicians and patients. This might have selected particularly motivated participants, and the results may not apply to the general population. Cost-efficacy estimation was not performed because this project was free of charge for health system and for the patients.

## Conclusions

The present study demonstrated that a health program based on GPs empowerment and taking care plus remote consultation by Consultants is at least as effective as standard outpatient management, in order to improve the control of type 2 diabetes and of some other features of metabolic syndrome.
